# Different health systems – Different mortality outcomes? Regional disparities in avoidable mortality across German-speaking Europe, 1992–2019

**DOI:** 10.1016/j.socscimed.2023.115976

**Published:** 2023-07

**Authors:** Michael Mühlichen, Mathias Lerch, Markus Sauerberg, Pavel Grigoriev

**Affiliations:** a Federal Institute for Population Research (BIB), Friedrich-Ebert-Allee 4, 65185, Wiesbaden, Germany; b Swiss Federal Institute of Technology in Lausanne (EPFL), Route Cantonale, 1015, Lausanne, Switzerland

**Keywords:** Amenable mortality, Preventable mortality, Spatial differences, Long-term trends, Cause-deleted life tables, German-speaking Europe

## Abstract

**Background:**

Evaluating the impact of health systems on premature mortality across different countries is a very challenging task, as it is hardly possible to disentangle it from the influence of contextual factors such as cultural differences. In this respect, the German-speaking area in Central Europe (Austria, Germany, South Tyrol and large parts of Switzerland) represents a unique ‘natural experiment’ setting: While being exposed to different health policies, they share a similar culture and language.

**Methods:**

To assess the impact of different health systems on mortality differentials across the German-speaking area, we relied on the concept of *avoidable mortality*. Based on official mortality statistics, we aggregated causes of death below age 75 that are either 1) amenable to health care or 2) avoidable through primary prevention. We calculated standardised death rates and constructed cause-deleted life tables for 9 Austrian, 96 German, 1 Italian and 5 Swiss regions from 1992 to 2019, harmonised according to the current territorial borders.

**Results:**

There are strong north-south and east-west gradients in amenable and preventable mortality across the studied regions to the advantage of the southwest. However, the Swiss regions still show significantly lower mortality levels than the neighbouring regions in southern Germany. Eliminating avoidable deaths from the life tables reduces spatial inequality in life expectancy in 2017/2019 by 30% for men and 28% for women.

**Conclusions:**

The efficiency of health policies in assuring timely and adequate health care and in preventing risk-relevant behaviour has room for improvement in all German regions, especially in the north, west and east, and in eastern Austria as well.

## Highlights

• Strong north-south and east-west gradients across the studied area.
• Swiss regions and South Tyrol show the lowest outcomes of avoidable mortality.
• Even best-performing regions of Germany and Austria are above Swiss level.
• Spatial pattern related to varying effectiveness of health care and health policies.
• Avoidable deaths explain approx. 30% of regional variation in life expectancy.

## 1 Background

The concept of ‘avoidable mortality’ is widely used to estimate the impact of health care and health policies on premature mortality (Nolte et al., 2002; Gianino et al., 2017). In cross-country comparisons, it is however hardly possible to separate this effect from the impact of other contextual factors, especially with regard to cultural differences (Mühlichen, 2019). For this reason, neighbouring regions in bordering countries that share a common or similar culture are eminently suitable for studying avoidable mortality.

The German-speaking regions in Central Europe are an extraordinarily interesting case, as while they share a common language and have substantial cultural, historical and economic ties, the populations are nonetheless divided by national borders and exposed to different health systems. Moreover, the respective countries show similar systems of federalism, in which the Austrian and German federal states, the northern Italian South Tyrol region and the Swiss cantons have a certain degree of autonomy, partly resulting in varying health policies even on a regional level (Lehmbruch, 2019). Therefore, the comparative study of avoidable mortality across the German-speaking regions can be seen as a unique ‘natural experiment’ (cf. Vaupel et al., 2003; Vogt and Vaupel, 2015).

In all German-speaking regions, inhabitants benefit from mandatory universal health insurance that provides access to essential health care, and health expenditures are exceptionally generous in international comparison, particularly in Germany and Switzerland (Busse et al., 2017; Bachner et al., 2018; OECD, 2021; Jasilionis et al., 2023). Yet, there are still substantial mortality differences across the German-speaking area. Whereas Switzerland is among the global vanguard countries that show an exceptionally high life expectancy at birth (83.79 years in 2019 for both sexes combined), Germany (81.20 years) and Austria (81.91 years) lag behind (all values taken from the Human Mortality Database, HMD, 2022). Nonetheless, there is considerable regional variation in life expectancy in Germany – to the disadvantage of the east and north – with the federal states of Baden-Württemberg and Bavaria in the south showing life expectancy values that are closer to Swiss and Austrian levels (82.13 and 81.90 years based on our calculations according to the HMD methodology).

Even though these striking regional mortality disparities might be associated with differences in national health systems and/or regional health policies, this has not been examined systematically for the three countries so far. Previous studies on avoidable mortality estimated differences between Austrian federal states in 2018 (Hofmarcher and Singhuber, 2020), sub-national trends in Germany (Sundmacher, 2013; Mühlichen, 2019), and differences between Swiss language regions (Feller et al., 2017), but these studies used different classifications of avoidable mortality and different periods.

Aiming to assess the impact of differences in health systems and/or health policies on spatial mortality variation across the three countries for the first time, we calculate standardised death rates of avoidable mortality across the German-speaking regions for the period from 1992 to 2019. To account for different risk factors, we divided avoidable causes of death into those that are 1) amenable to health care and those that are 2) avoidable through primary prevention. Through the application of cause-deleted life tables, we estimate the effect of avoidable deaths on regional outcomes of life expectancy. The chosen regional level of overall 111 spatial units allowed us to discover if a geographical continuum of mortality from north to south exists or if there are still systematic differences along the national borders.

## 2 Data and methods

### 2.1 Selected area

For regional differentiation, we chose Austria's 9 federal states (‘Bundesländer’), Germany's 96 spatial planning regions (‘Raumordnungsregionen’), one Italian province (South Tyrol) and 5 Swiss major regions (‘Grossregionen’) because they are the most comparable subnational divisions in terms of structure and population size. The German spatial classification is taken from the Federal Institute for Research on Building, Urban Affairs and Spatial Development (BBSR, 2017). The Austrian, Italian and Swiss spatial units are based on the NUTS-2 classification (Eurostat, 2021). We excluded the predominantly French- and Italian-speaking NUTS-2 regions in western and southern Switzerland (Lake Geneva region and Ticino) from analysis. However, an exact separation of French- and Italian-speaking areas is not entirely possible by using administrative divisions. For instance, the mostly German-speaking capital region surrounding Bern includes French-speaking areas in the west, whereas the excluded Lake Geneva region includes the German-speaking Upper Wallis area in the east. We did not consider additional German-speaking countries or regions because they are comparatively small and do not have comparable time series for cause-specific mortality available. Fig. 6 in the appendix shows a map of the selected regions.

### 2.2 Data selection and preparation

We collected official data on causes of death and population size by sex, age (0, 1–4, 5–9, …, 85–89, 90+) and region from the statistical offices of Austria, Germany, Italy and Switzerland. Causes of death have been recorded according to the 10th International Classification of Diseases (ICD-10) since 1995 in Switzerland, 1998 in Germany, 2002 in Austria and 2003 in Italy. In earlier years, Germany, Italy and Austria used ICD-9, whereas Switzerland used ICD-8 before 1995. We chose 1992 as the starting year for the analysis, because this is the first year that cause-of-death time series is available for all regions in Germany.

To account for the census break, we harmonised population counts in Germany before 2011 by redistributing the differences in 2011 between the first published age- and sex-specific numbers and the census-corrected ones proportionally over the previous intercensal years (cf. Klüsener et al., 2018). This adjustment was necessary because the 2011 Census revealed official counts were overestimated by 1.5 million residents. Furthermore, we applied the current territorial borders of the study regions to all years of observation, allowing for a completely harmonised long-term cause-specific time series, which has not been done previously for Germany on a regional level smaller than the 16 federal states.

### 2.3 Avoidable mortality

The concept of avoidable mortality, first developed by Rutstein et al. (1976), classifies causes of death into causes amenable to health care (‘amenable’ or ‘treatable’ mortality) and causes avoidable through primary prevention (‘preventable’ mortality). Amenable mortality is an indicator of the effectiveness of health care through secondary prevention or medical treatment, whereas preventable mortality is an indicator of the effectiveness of inter-sectoral health policies in the broad sense and largely reflects risk-relevant behaviour of the population, e.g. smoking and alcohol abuse (Nolte et al., 2002). We aggregated cause-specific deaths into the groups of amenable and preventable causes according to the classification introduced by Mühlichen (2019) – which is widely based on Nolte and McKee (2004, 2012) for amenable mortality and Page et al. (2006) for preventable mortality – with slight adjustments to make it compatible for ICD-8 through 10. In line with most concepts of premature mortality, we did not classify any deaths from age 75 upwards as avoidable, “as ‘avoidability’ of death and reliability of death certification become increasingly questionable at older ages” (Nolte and McKee, 2004, p. 65). Table 2, Table 3 in the appendix show our applied cause-of-death grouping in detail.

### 2.4 Methods

To avoid distortion by compositional differences or changes in the regional populations, we calculated standardised death rates (SDR) for 3-year periods using a directly standardised age and sex structure for each region (according to the 2013 European Standard Population). This allows direct comparison of the differences in amenable and preventable mortality over time and between regions and sex.

In addition, to estimate the contribution of avoidable causes on regional mortality disparities, we applied cause-deleted life tables (Keyfitz and Caswell, 2005, pp. 42–45; Beltrán-Sánchez et al., 2008) for the same 3-year periods by sex and region. While the conventional life table produces an estimate of life expectancy at birth based on age-specific death counts observed in a given population, cause-deleted life tables refer to the hypothetical situation in which specific causes of death are removed from the observed set of age-specific death counts. We compared life expectancy at birth in the study regions before and after removing amenable and preventable deaths from the age-and sex-specific death counts. To assess the extent of regional differences before and after this removal, we estimated statistical summary measures: minimum, maximum, mean and standard deviation (SD), the latter two both unweighted and weighted by population size.

We conducted all calculations in R (R Core Team, 2023), and we used ArcMap (Esri, 2020) to merge the NUTS-2 shapefile from Eurostat (2021) with the German shapefile from the Federal Agency for Cartography and Geodesy (BKG, 2022) and construct the maps for this paper.

## 3 Results

### 3.1 Trends in avoidable mortality

**Fig. 1:**
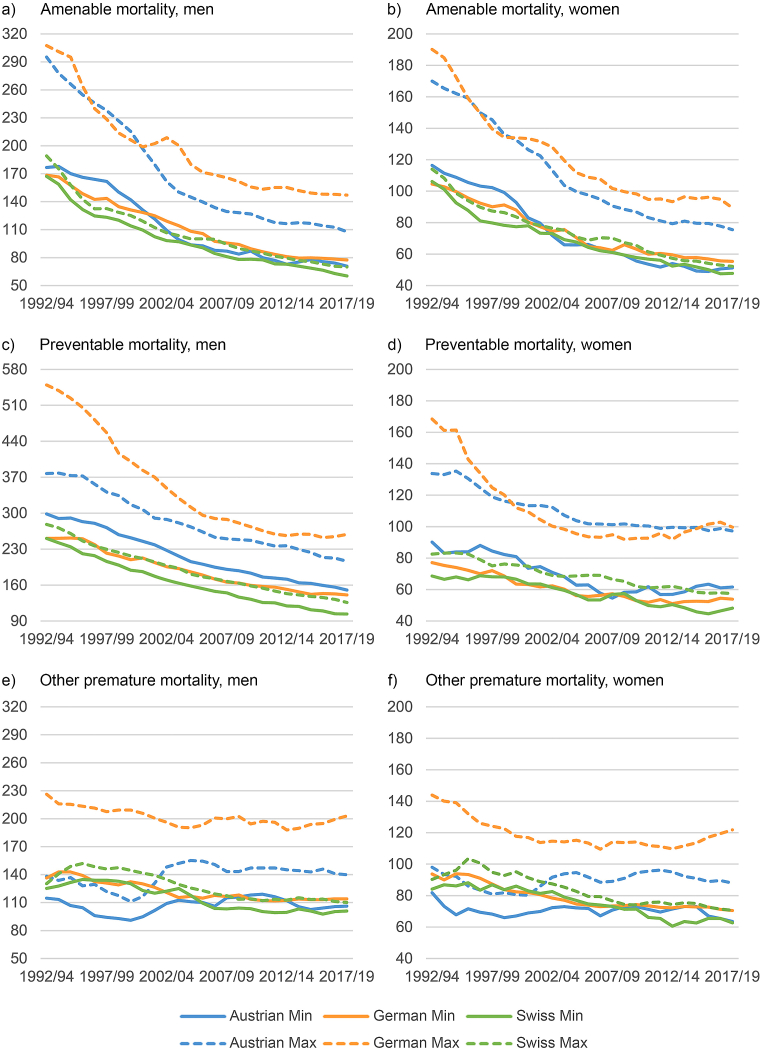
Amenable and preventable mortality in Austrian, German and Swiss regions by sex from 1992/1994 to 2017/2019 (moving averages); standardised death rates (deaths per 100,000 inhabitants); minimum and maximum values by country.

Fig. 1 shows the long-term trends in amenable, preventable and other premature mortality for men and women in Austria, Germany and Switzerland from 1992/1994 to 2017/2019. For visibility reasons, we only show the regional minimum and maximum values by country for each 3-year period and use different y-scales for each panel of the graphic. The trends in selected regions, including South Tyrol, are shown in Fig. 7 in the appendix. Fig. 1, Fig. 7 reveal enormous decreases over time in the three countries, which, in relative terms, were higher in amenable mortality than in preventable mortality. South Tyrol and the Swiss regions exhibited the lowest values of amenable and preventable mortality almost consistently throughout the observation period. Due to the small regional differences in Switzerland, even the country's maximum values were at times slightly lower than the German and Austrian minimum values in male amenable and preventable mortality, e.g. in the most recent years of the analysis. German regions, on the contrary, largely showed the highest values in amenable, preventable and other premature mortality. While differences in amenable mortality, for both men and women, between the best-performing regions of the three countries were small in the early 1990s, the Swiss advantage increased considerably in the mid-1990s, which was followed by a period of convergence from the late 1990s, then a re-expansion of the gap after 2014. Considering preventable mortality, the differences between the countries' best-performing regions remain largely constant from the mid-1990s, interrupted by a phase of convergence among women in the 2000s. The enormous decreases in German maximum values in the 1990s in both amenable and preventable mortality – and to a lesser extent in other premature mortality – were driven by eastern Germany catching up to the west following reunification (see Fig. 7). In general, the mortality levels and the regional differences were smaller among women than among men.

In other premature mortality, there was considerably less decline over time. In Austria, which performed best in this regard in the 1990s, there was even an upward shift in the 2000s, particularly among men, after which South Tyrol became the best-performing region. Several northwestern and eastern German regions also experienced an upward shift in the most recent years of observation. Regional differences are slightly smaller in other causes than in avoidable causes. Austrian, Swiss and southern German regions show similar rates, e.g. the German region of Munich is on the same level like the Swiss capital region in both sexes (see Fig. 7). However, beginning in the mid-2000s, there has been a growing advantage in South Tyrol and Switzerland.

### 3.2 Spatial patterns of avoidable mortality

**Fig. 2:**
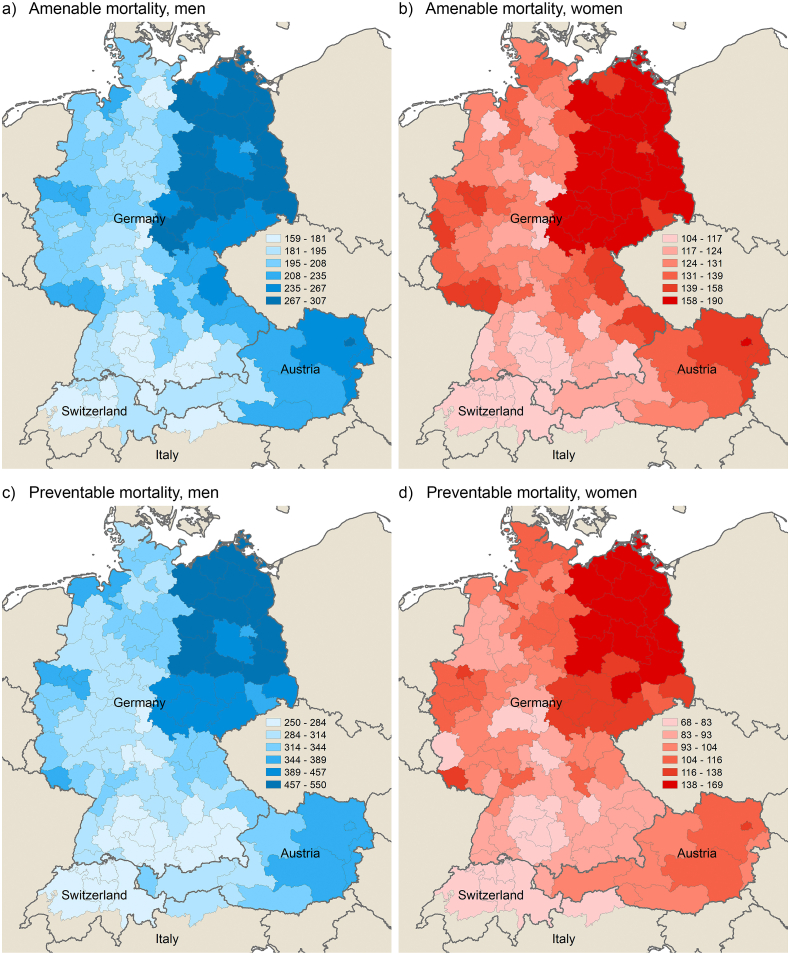
Amenable and preventable mortality across German-speaking regions by sex in 1992–1994; standardised death rates (deaths per 100,000 inhabitants).

**Fig. 3:**
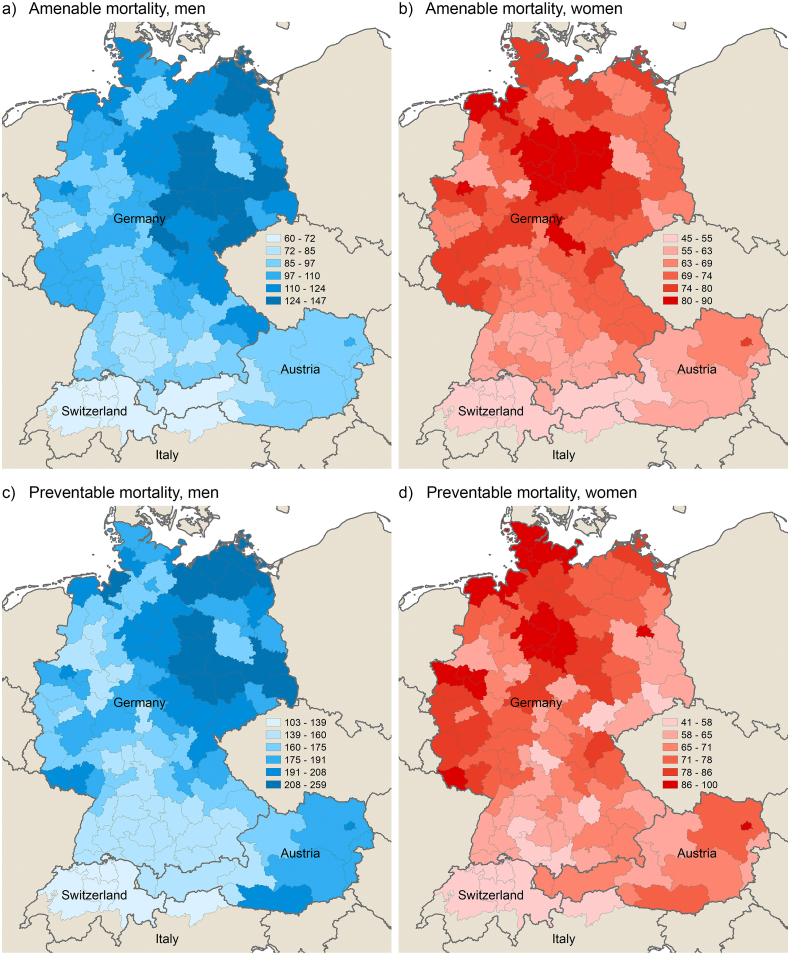
Amenable and preventable mortality across German-speaking regions by sex in 2017–2019; standardised death rates (deaths per 100,000 inhabitants).

Fig. 2, Fig. 3 show the spatial distribution of amenable and preventable mortality across the study area in 1992–1994 and 2017–2019, respectively (men on the left and women on the right). Fig. 8 in the appendix shows the spatial patterns for other premature mortality.

In Germany, there are both east-west and north-south gradients, with more intensity in the former among men, and more prevalent in the latter among women, particularly in preventable mortality. Whereas the east-west gap significantly decreased over time, the magnitude of the north-south gradient increased. Although the southern German regions have significantly lower rates of amenable and preventable mortality than the northern and eastern part of Germany, they are still lagging behind the neighbouring regions in western Austria and Switzerland. The southern German metropolitan areas of Munich and Stuttgart have been consistently among the best-performing regions within Germany over the study period, yet they showed higher rates compared to the Swiss regions in most years of observation, with the gap even increasing slightly in male preventable mortality in the last years of the study period (as shown more explicitly in Fig. 7). An exception to these overall patterns is the Dresden agglomeration in Saxony, in which preventable female mortality has increasingly caught up to Swiss levels from the mid-2000s (as also shown in Fig. 7).

In Austria, there is a strong east-west gradient with the highest rates of amenable and preventable mortality in the capital of Vienna. In fact, preventable female mortality was highest in Vienna among all study regions for most of the observed years. Tyrol – a mountainous area in the west of the Austria – was consistently among Austria's best-performing regions and close to Swiss levels in amenable mortality.

South Tyrol, the Italian region bordering Tyrol to the north and east, showed the highest life expectancy at birth of all studied regions for both men and women in the most recent years of observation. Whereas amenable mortality was on a similar level to Tyrol and Switzerland, South Tyrol showed particularly low rates in preventable mortality and other premature mortality.

In Switzerland, there are no outstanding regional differences. The central part of Switzerland surrounding the city of Luzern showed particularly low mortality rates, whereas the capital region in the west – that includes not only the Bern agglomeration but also economically peripheral regions in the northwest and southwest – and the mountainous, peripheral east of Switzerland mostly showed higher rates than the other Swiss regions.

Overall, there is a smoother continuum from southern Germany to Austria and Switzerland in the spatial distribution of amenable mortality than preventable mortality in the early 1990s. However, in the period from 2017 to 2019, this continuum appears more evident in preventable mortality, while the advantage of Switzerland, South Tyrol and, to a lesser degree, western Austria, has grown in amenable mortality relative to the other regions.

### 3.3 Cause-deleted life tables

**Fig. 4:**
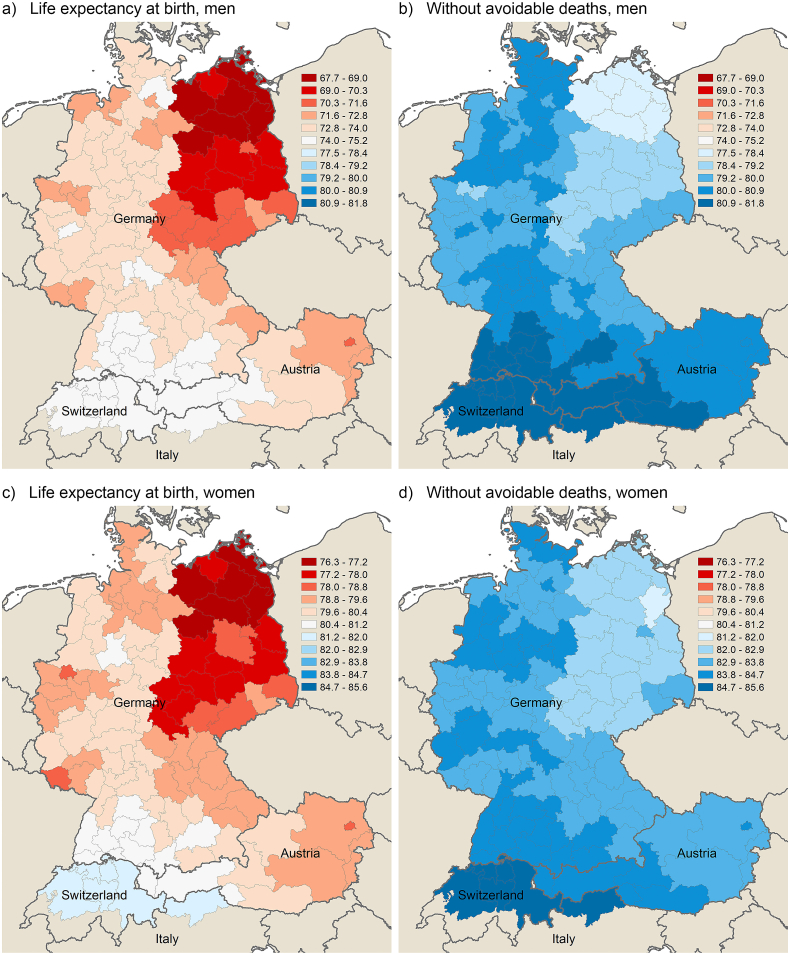
Life expectancy at birth before and after removing avoidable deaths from life tables across German-speaking regions by sex in 1992–1994. Note: Same scale used for panels a and b (men) and for panels c and d (women) to make differences clearer. In panels a and b, there are no regions in the range from 75.2 to 77.5.

**Fig. 5:**
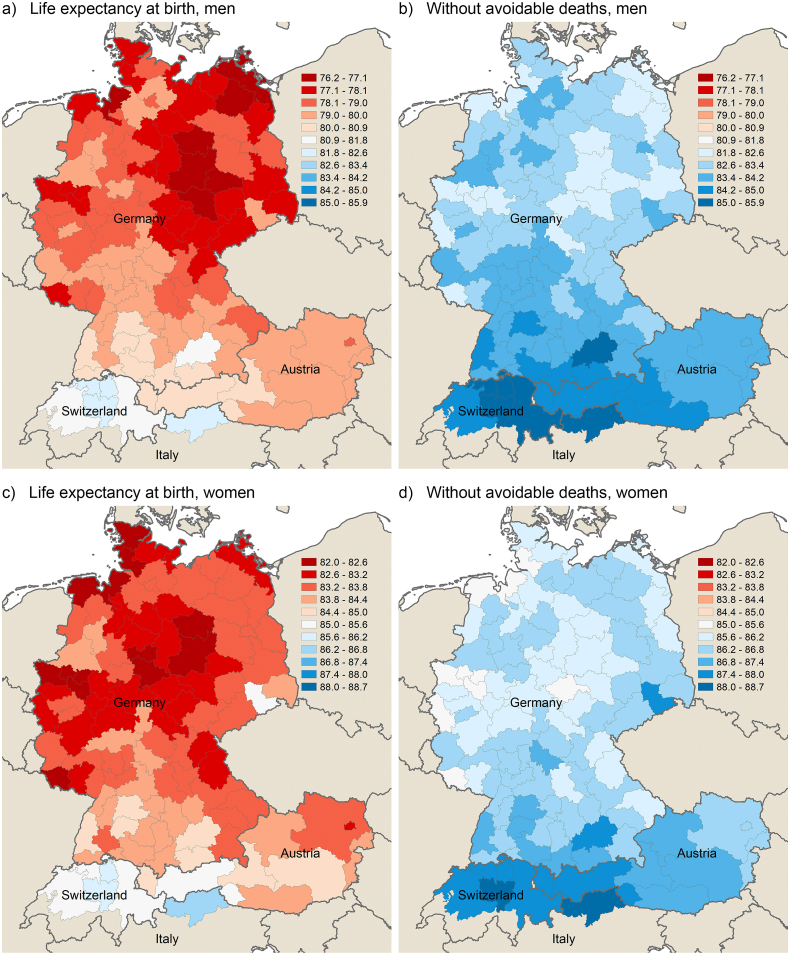
Life expectancy at birth before and after removing avoidable deaths from life tables across German-speaking regions by sex in 2017–2019. Note: Same scale used for panels a and b (men) and for panels c and d (women) to make differences clearer.

**Fig. 6:**
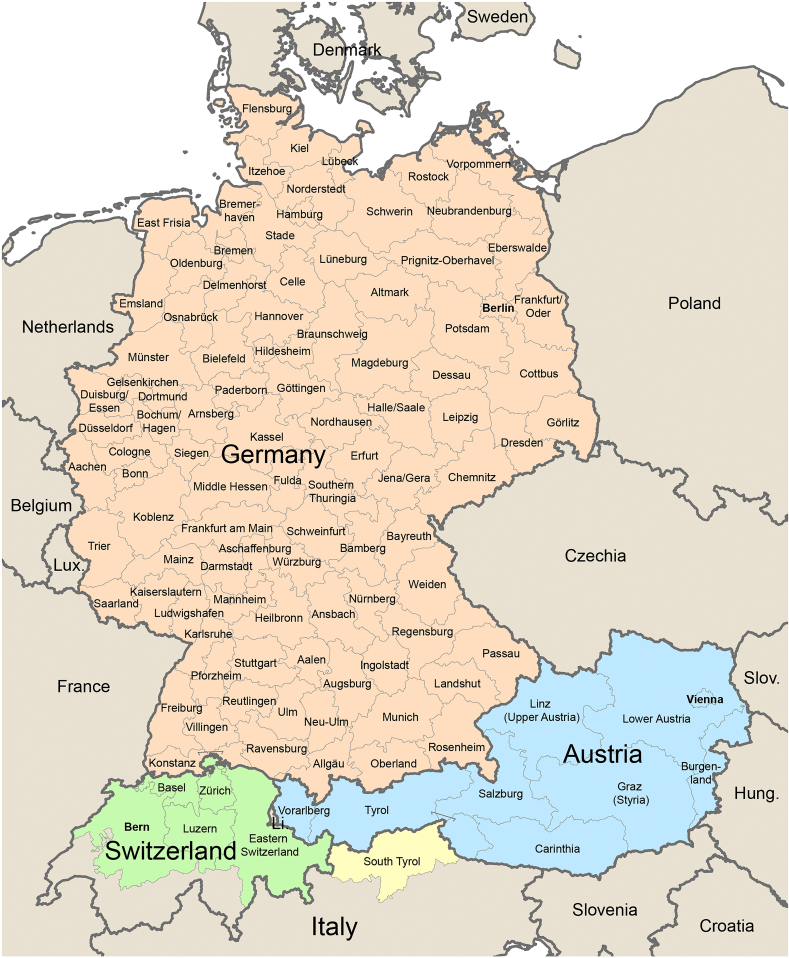
Selected regions: 9 Austrian federal states (‘Bundesländer’), 96 German spatial planning regions (‘Raumordnungsregionen’), 1 Italian province and 5 Swiss major regions (‘Grossregionen’) Note: For visibility, we mostly used the names of the central cities instead of the regions' names. Abbreviations: Hung. = Hungary; Li. = Liechtenstein; Lux. = Luxemburg; Slov. = Slovakia.

**Fig. 7:**
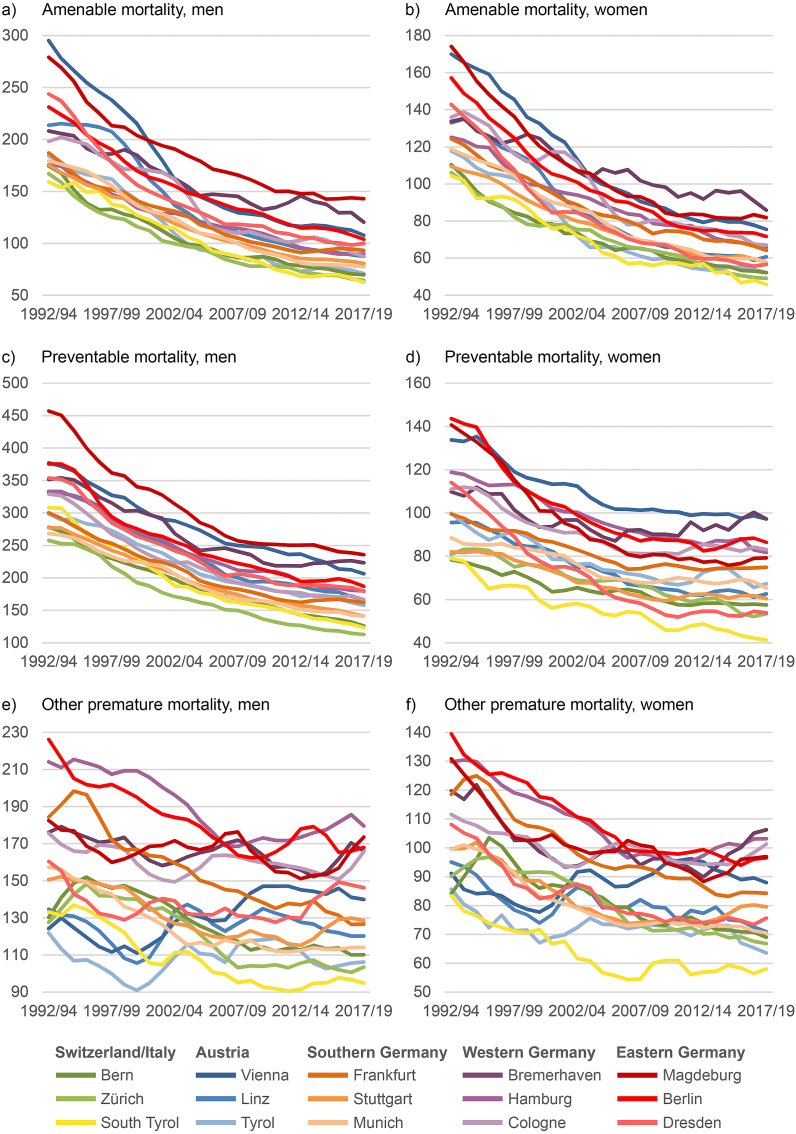
Amenable and preventable mortality in selected Austrian, German and Swiss regions and South Tyrol by sex from 1992/1994 to 2017/2019 (moving averages); standardised death rates (deaths per 100,000 inhabitants). Notes: For visibility, we dropped confidence intervals and used different y-scales for each panel. We chose regions that represent different geographical areas and different mortality levels.

**Fig. 8:**
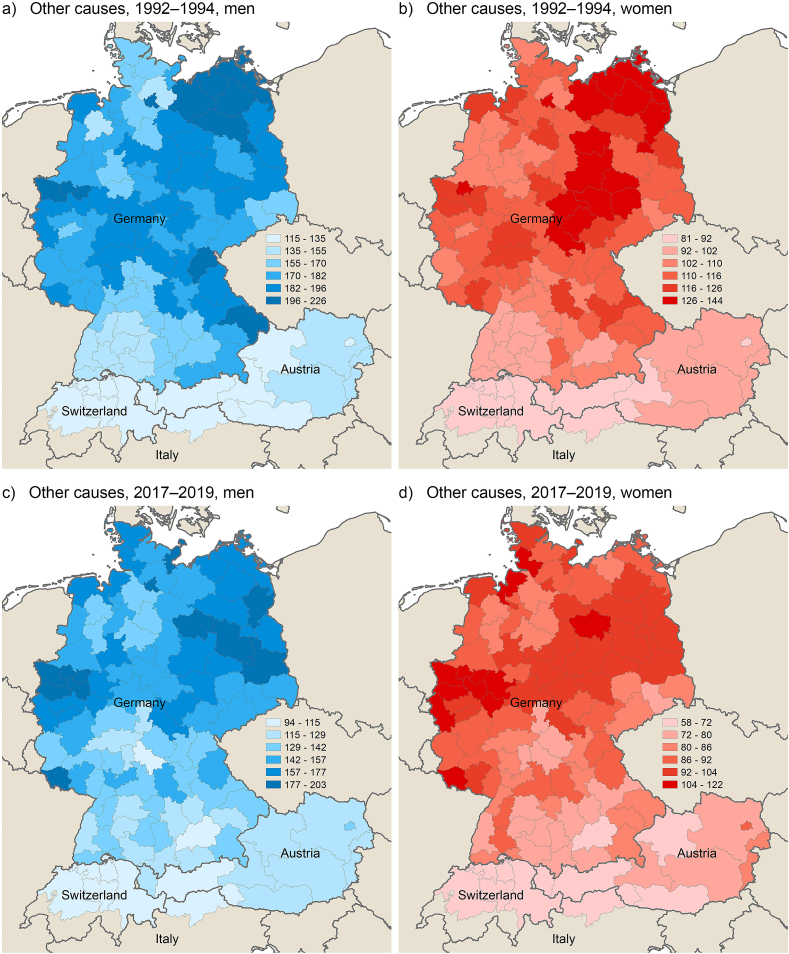
Premature mortality from ‘non-avoidable’ causes across German-speaking regions by sex in 1992–1994 and 2017–2019; standardised death rates (deaths per 100,000 inhabitants).

To quantify the role of avoidable deaths in the spatial context of overall mortality, Fig. 4, Fig. 5 illustrate the regional pattern of life expectancy for men and women in 1992–1994 and 2017–2019, both before and after the elimination of avoidable deaths. The figures show that the variation in life expectancy at birth decreases after the elimination of avoidable deaths (amenable and preventable deaths combined) for both men and women. Especially the German east-west gradient loses significance, whereas the north-south gradient remains for the major part.

In more detail, the weighted standard deviation (SD) decreases from 1.30 to 0.91 for men (by 30%) and from 0.90 to 0.65 for women (by 28%) in 2017–2019, signalling a considerable decrease in spatial inequality when avoidable deaths are not considered (Table 1). The range between minimum and maximum values decreases from 6.19 to 4.07 among men and from 4.70 to 3.49 among women. Concerning the earlier period, the relative reduction of regional disparities in life expectancy by eliminating avoidable deaths is even higher: the weighted SD decreases by 43% among men (from 1.49 to 0.85) and by 31% among women (from 1.09 to 0.75), which is related to the comparatively high level of avoidable mortality in eastern Germany in the early 1990s.

**Table 1:** Life expectancy at birth before and after elimination of avoidable deaths; summary measures for 111 German-speaking regions by sex in 1992–1994 and 2017–2019.

		1992–1994		2017–2019	
		*LE at birth*	*Without AD*	*LE at birth*	*Without AD*
***Men***	Minimum	67.67	77.50	76.19	81.82
	Maximum	75.21	81.80	82.38	85.89
	Mean	72.75	80.01	78.87	83.39
	Weighted mean	72.89	80.04	79.09	83.51
	SD	1.70	0.89	1.30	0.87
	Weighted SD	1.49	0.85	1.30	0.91
***Women***	Minimum	76.38	81.76	81.98	85.21
	Maximum	81.89	85.53	86.68	88.70
	Mean	79.45	83.64	83.57	86.39
	Weighted mean	79.51	83.71	83.64	86.46
	SD	1.16	0.78	0.88	0.63
	Weighted SD	1.09	0.75	0.90	0.65

Notes: SD is standard deviation, LE is life expectancy. Avoidable deaths (AD) include both amenable and preventable deaths. Weighted measures were weighted by population size.

As additional analyses reveal, the remaining regional mortality differences – including the reduced but still-evident advantage of Switzerland and South Tyol – are mostly due to differences at ages 75 and older which are not addressed by the concept of avoidable mortality.

## 4 Discussion

The results of this study indicate that northern and eastern Germany as well as eastern Austria have room for improvement in providing adequate and timely medical care and in reducing risk-relevant behaviour. In comparison, Switzerland and South Tyrol show considerably lower rates of amenable and preventable mortality. Even though the regional patterns in amenable and preventable mortality reveal a continuum – at least in part – from north to south and east to west, the southern borders to Switzerland and South Tyrol still make a systematic difference.

### 4.1 Sex differences

Mortality rates are generally higher and regional differences more pronounced among men than among women. The sex differences are particularly large for preventable mortality. However, one important result of this study is that the extent of spatial inequality would be on a similar level for both sexes if avoidable deaths were eliminated, even though overall mortality would be still higher among men. In general, men's mortality levels are more sensitive to their social and economic environment as compared to women due to higher labour force participation in the context of traditional social roles (McKee and Shkolnikov, 2001; Oksuzyan et al., 2014). Overall, men were found to show higher smoking rates and are less likely to engage in health behaviours associated with primary prevention (Valkonen and van Poppel, 1997; Trovato, 2005; Hiller et al., 2017).

### 4.2 Germany

In Germany, the ‘traditional’ east-west gap in amenable mortality has decreased significantly for both sexes and is only still evident among men. The lower level of amenable cancer mortality among eastern German women is particularly related to lower death rates from breast cancer, which is likely related to lower childlessness in the relevant cohorts born before reunification and to the vanguard position – especially in Saxony – in establishing cancer registries, including certified tumour centres/boards (Westerman and Mühlichen, 2019). From a historical viewpoint, individuals living in eastern Germany were exposed to a less efficient health care system than Germans living in the west before German reunification (Grigoriev and Pechholdová, 2017; Grigoriev et al., 2021). The differences in the effectiveness of preventive health care due to the lack of sophisticated medical equipment and modern technologies in the east have resulted in higher mortality rates from cardiovascular diseases and cancers in regions located in the former German Democratic Republic (GDR) (Nolte et al., 2002). While the impact of those worse conditions during the GDR era on current mortality outcomes continues to decline, access to timely medical care is still a challenge in several peripheral areas of the east, intensified by selective emigration (Vogt and Vaupel, 2015; Mühlichen, 2019). For instance, the rural areas of eastern Germany show longer distances to health care facilities and a lower physician density per people of retirement age (BBSR, 2022). Sundmacher and Ozegowski (2016) have shown that the spatial distribution of ambulatory specialists is associated with the proportion of privately insured individuals, and thus more concentrated in socioeconomically advanced regions.

There is also a north-south gradient in amenable mortality among both sexes, which is likely related to economic and structural advantages in southern Germany, fostered by selective immigration (cf. Kibele et al., 2015). This is particularly true for metropolitan areas, such as Munich and Stuttgart, but there are also rapid-growing areas in other parts of the country that show low levels of amenable mortality, such as Hamburg, Potsdam, Dresden, Münster and Bonn. In general, the German pattern of higher mortality in the north and east is highly connected with socioeconomic conditions, particularly among men (Kibele, 2012; Kibele et al., 2015; Rau and Schmertmann, 2020; Michalski et al., 2022; Hrzic et al., 2023). In the context of amenable mortality, socioeconomic conditions on the macro level affects the availability of adequate health care, while socioeconomic status on the individual level influences the utilisation of these services and health-related behaviour in general (Kibele, 2012; Lampert et al., 2018; Wenau et al., 2019; Tetzlaff et al., 2020; Van Raalte et al., 2020).

In preventable mortality, the east-west gap has also decreased considerably. However, among men, it is still the most visible regional divide, whereas it has completely disappeared among women, to the disadvantage of the northwest (from Saarland to Flensburg). In eastern Germany, the consequences of the political, economic and social transformation crisis following reunification have gained in importance among men, as the cohorts that were most affected by long-term unemployment and disadvantageous working conditions – particularly men born between 1950 and 1985 – increasingly reach the mortality-relevant age (Klüsener et al., 2020; Grigoriev et al., 2021). In addition, the east has experienced a long stage of selective emigration (‘brain drain’), which intensified the disadvantages in socioeconomic factors (Heiland, 2004; Luy and Caselli, 2007; Westphal, 2016; Stawarz and Sander, 2020). People living in socioeconomically deprived areas show higher risks of smoking, alcohol abuse and suicides as compared to inhabitants of areas with a stable economic situation (Kleinschmidt et al., 1995; Jasilionis et al., 2020; Jakobsen and Lund, 2022).

The German regional pattern for preventable mortality is particularly connected with smoking. Just like in amenable mortality, there is also a north-south gradient in preventable mortality, to the advantage of the south, including – at least among women – the south of eastern Germany, too. Recent analyses of the German Microcensus 2017 confirm that smoking rates are considerably lower in these areas compared to the north and west of Germany (Mühlichen et al., 2023). Accordingly, previous research has shown that regional differences in smoking histories explain large parts of the observed spatial mortality patterns in Germany (Grigoriev et al., 2022; Vogt et al., 2017). Among the birth cohorts that were below age 45 in 2017, however, the east shows the highest smoking rates among both men and women (Mühlichen et al., 2023), thus fuelling worries about a rewidening of the east-west gap as soon as these cohorts reach the mortality-relevant age (Myrskylä and Scholz, 2013; Vogt et al., 2017).

### 4.3 Austria

Austria shows a clear east-west gradient in both, amenable and preventable mortality with higher mortality levels observed in the east of Austria. Similar to Germany, the Austrian regions located in the west, such as Tyrol and Vorarlberg, are not only characterized by lower avoidable mortality rates but also show the highest Gross Domestic Product (GDP) per capita (Statistics Austria, 2023). In particular, the capital city of Vienna stands out as a high-mortality region. Thus, distance to health care facilities might not play the most important role for explaining Austria's spatial pattern in terms of amenable mortality. Given that Austria is among the most generous countries in terms of health expenditures in the EU – even though still below the German and Swiss levels – and shows an exceptionally high physician density, particularly in urban areas, regional differences in the utilisation of health care services might contribute more to disparities in health-related outcomes (Bachner et al., 2018; Renner, 2020). It should be noted, for instance, that the population of Vienna is heterogenous with respect to socioeconomic status, which affects individual's utilisation of medical care (Renner, 2020). Accordingly, previous research has focused mostly on regional disparities in socioeconomic and behavioural factors, such as smoking, drinking and diet (Rau et al., 2008; Klotz and Doblhammer et al., 2008; Klimont, 2010; Stein et al., 2011).

The level of alcohol consumption is particularly high in Vienna and Burgenland, while people in Vorarlberg are drinking comparatively less (Uhl et al., 2015). As a result, alcohol-attributable mortality is lower in the west of Austria compared to the east, which holds especially among men (Urbas et al., 2007). Our results for preventable mortality are in line with these findings, highlighting the connection between health behaviour and the comparatively high mortality level in the east. Along with alcohol consumption, cigarette smoking plays an important role for assessing regional mortality differentials in Austria. Stein et al. (2011) found a clear east-west gradient in cardio-vascular mortality in Austria, which can be partly explained by coronary risk factors like smoking. Since health behaviours are usually strongly associated with socioeconomic factors such as education, some of the observed differences in regional mortality are likely linked to differences in the population composition by educational attainment (Rau et al., 2008; Klotz and Doblhammer, 2008).

### 4.4 South Tyrol

South Tyrol is a widely autonomous province in the north of Italy that formerly belonged to Austria until 1919 and still has a German-speaking majority and close cultural and economic ties to its northern neighbouring country (Grote, 2007). The widely rural and mountainous region has become an exceptionally wealthy region in recent decades showing an outstandingly high life expectancy in national and international comparison (Woelk, 2017; Petrelli et al., 2019; Eurostat, 2023). In our study, South Tyrol was consistently among the best-performing regions in amenable and preventable mortality along with central Switzerland. However, in amenable mortality, the gap between South Tyrol and Tyrol, its Austrian neighbouring region, was very small. Aside from its favourable economic conditions, South Tyrol shows comparatively low rates of smoking, whereas the physician density is lower as compared to Austria (Bachner et al., 2018; ASTAT, 2023).

### 4.5 Switzerland

There is no clear regional gradient in Switzerland. Among the German-speaking regions, we found the lowest levels of amenable and preventable mortality in central Switzerland surrounding the city of Luzern, closely followed by the regions of Zurich and Basel. With the exception of the highly urbanised Zurich region, the Swiss major regions are composed by a heterogeneous set of subregions in terms of economic development and level of urbanisation (especially the capital region surrounding Bern). In amenable mortality, Eastern Switzerland and Bern in the central west show the highest rates. The Swiss health infrastructure is highly developed and of similar quality across the whole country, except for some isolated alpine areas, even though the Swiss health care system is highly federalised on the canton level (Gygli et al., 2021; Schusselé Filliettaz et al., 2021). The small observed regional gradients in amenable mortality may thus be essentially related to regional differences in the utilisation of health care services (Camenzind, 2012; Marques-Vidal and Paccaud, 2012).

In preventable mortality, regional differences are small and widely show the same gradients as in amenable mortality. Differences in health behaviours are rather limited across Switzerland's major regions and are more pronounced between the three linguistic zones and between urban and rural areas (Bopp and Gutzwiller, 1999; Wanner et al., 2012; Lerch et al., 2017). Generally, the German-speaking regions of Switzerland are characterized by low levels of smoking and alcohol consumption and high frequency of physical activity (Wanner et al., 2012; Boes et al., 2016).

### 4.6 Limitations

In general, there is no perfect classification of avoidable mortality, and (sub-)national differences in cause-specific mortality can be influenced by different coding practices (Mackenbach et al., 2013). To minimise potential distortions of regional differences due to varying degrees of coding accuracy, we have used a classification that considers broader causal groups based on 3-digit ICD codes instead of very specific 4-digit codes. Our analyses indicate that the improvements in overall premature mortality are closely connected with the decreases in amenable and preventable mortality. ‘Non-avoidable’ premature deaths showed comparatively small decline and – even though the spatial gradients were similar – smaller regional variation, which points to the suitability of the used cause-of-death classification. Despite this, we cannot rule out entirely that differences in coding practices might have an effect on our results. For instance, the Austrian mortality levels in ‘non-avoidable’ premature mortality were comparatively low in the 1990s but shifted upwards following the introduction of ICD-10 in 2002, while at the same time, amenable mortality shifted downwards. The same phenomenon – but to a lesser extent – can be seen for Switzerland following the change from ICD-8 to ICD-10 in 1995.

In addition, the selected regional classification does not account for urban-rural differences, as urban areas are grouped together with their surroundings (except for Berlin, Bremen, Hamburg and Vienna). Urban-rural differences partly explain regional differences in mortality between eastern and western Germany (Mühlichen, 2019; Ebeling et al., 2022). In Switzerland, regional differences in mortality faded out at the turn of the 21st century, while mortality gradients according to the urban-rural continuum of space gained importance (Wanner et al., 2012; Lerch et al., 2017). For this paper, however, it was our priority to analyse regions that are of comparable size and structure, also because health systems rather vary by region, not by the degree of urbanisation. However, the latter influences access to adequate and timely health care (Mühlichen, 2019; Ebeling et al., 2022). Nonetheless, there is no internationally comparable urban-rural classification so far and the varying administrative regional divisions make it difficult to separate urban from rural areas in a consistent way.

## 5 Conclusion and outlook

The results show that the lower levels of life expectancy in Germany – and generally in the north and east of the German-speaking area – are probably associated with lacking efficiency of health policies in assuring timely and adequate medical care and in preventing risk-relevant behaviour, including non-optimal use of these services by the population. However, the elimination of avoidable deaths from the life tables reduces the north-south gradient only moderately, pointing to the significance of mortality differences at older ages, where the majority of deaths is concentrated (cf. Jasilionis et al., 2023).

Future research should focus on the single causes of death that drive these regional patterns – especially with regard to specific risk factors like smoking and alcohol abuse – and the impact of urban-rural differences as well as the driving factors for the regional mortality gradients at older ages in the three countries. In addition, ecological studies that link health outcomes with available contextual data are important for further in-depth insights, e.g. multi-level modelling controlling simultaneously for different sources of variability (individual and contextual effects).

## Author contributions

MM and ML collected the data. MM prepared the data, conceived, designed and performed the analyses, and wrote the initial draft of the paper. ML and MS contributed to the discussion. PG and MM wrote the R scripts for the statistical analyses. MS, ML and PG provided critical revisions of the manusript.

## Funding

This study has received funding from the 10.13039/501100000781European Research Council (ERC) under the European Union’s Horizon 2020 research and innovation programme (grant agreement No. 851485).

## Figures and Tables

**Table 2:** Causes considered amenable to health care^a^, ages 0–74 (unless otherwise stated).

Disease group	Cause of death	Age	ICD-8 code	ICD-9 code	ICD-10 code
*Infectious*	Intestinal infections	0–14	000–9	001–9	A00-9
	Tuberculosis		010–9	010-8, 137	A15–9, B90
	Whooping cough	0–14	033	033	A37
	Measles	1–14	055	055	B05
	Other infections		032, 034–8, 040–3, 084, 381–3, 681–2, 720	032, 034–8, 045, 084, 381-3, 681–2, 730	A35–6, A38–41, A46, A80, B50–4, H65–70, L03, M86, M89-90
*Neoplasm*	Colorectal cancer		153–4	153–4	C18-21
	Bone cancer		170	170	C40-1
	Skin cancer		172–3	172–3	C43-4
	Female breast cancer		174	174	C50
	Cervical cancer		180	180	C53
	Uterine cancer		182	179, 182	C54, C55
	Prostate cancer		185	185	C61
	Testicular cancer		186	186	C62
	Bladder cancer		188	188	C67
	Eye cancer		190	190	C69
	Thyroid cancer		193	193	C73
	Hodgkin's lymphoma		201	201	C81
	Leukaemia	0–44	204–7	204–8	C91-5
	Benign neoplasm		210–28	210–29	D10-36
*Endocrine*	Diseases of the thyroid		240–6	240–6	E00-7
	Diabetes mellitus (50%)	0–49	250	250	E10-4
*Neurological*	Bacterial meningitis		320	320	G00
	Epilepsy		345	345	G40-1
*Cardiovascular*	Rheumatic heart disease		390–8	390–8	I00-9
	Hypertensive disease		400–4	401–5	I10-3, I15
	Ischaemic heart disease (50%)		410–4	410–4	I20-5
	Heart failure^b^		427–9	428–9	I50-1
	Cerebrovascular disease (50%)		430–8	430–8	I60-9
*Respiratory*	Influenza		470–4	487–8	J09-11
	Pneumonia		480–6	480–6	J12-8
	Asthma	0–44	493	493	J45-6
	Other respiratory diseases	1–14	460-6, 500-519	460-79, 494–495, 497-519	J00–8, J20–39, J47-99
*Digestive*	Peptic ulcer		531–4	531–4	K25-8
	Appendicitis		540–3	540–3	K35-8
	Abdominal hernia		550–3	550–3	K40-6
	Cholelithiasis and cholecystitis		574–6	574–5	K80-2
*Genitourinary*	Nephritis and nephrosis		580-4, 792	580–9	N00–7, N17–9, N25-7
	Hyperplasia of prostate		600	600	N40
*Maternal/infant*	Maternal death		630–78	630–79	O00-99
	Perinatal deaths (excl. stillbirths)		760–79	760–79	P00–96, A33
	Congenital anomalies		740–59	740–59	Q00-99
*External*	Treatment complications		E930-6	E870-9	Y60-84

Notes.

a The list for amenable causes of death is based on Mühlichen (2019) who relied primarily on Nolte and McKee (2004, 2012) with slight adjustments done following Tobias and Jackson (2001), Page et al. (2006), Tobias et al. (2010) and Plug et al. (2011) (see Mühlichen, 2019, for details). Compared to Mühlichen (2019), we added prostate cancer due to advances in adjuvant therapy, including anti-androgens (following Tobias et al., 2010) and widened the age range for uterine cancer due to reasonable five-year relative survival (Page et al., 2006). In addition, we made minor, statistically negligible adjustments to allow for a more consistent cause-of-death coding from ICD-8 to ICD-10 (concerning influenza and other respiratory diseases).

b In ICD-8, it is not possible to identify heart failures on a three-digit level, at least not in the same way as in versions 9 and 10. The codes 427 to 429 include codes that are not directly related to heart failure, such as 427.2 (heart block). This is statistically negligible, though.

**Table 3:** Causes considered avoidable through primary prevention^a^; ages 0–74 (unless otherwise stated).

Disease group	Cause of death	Age	ICD-8 code	ICD-9 code	ICD-10 code
*Infectious*	Hepatitis		070	070	B15-9
	HIV/AIDS^b^		137	042–4	B20-4
	Sexually transmitted diseases		090–9	090–9	A50-64
*Neoplasm*	Cancer of lip, oral cavity, pharynx		140–9	140–9	C00-14
	Cancer of oesophagus		150	150	C15
	Cancer of stomach		151	151	C16
	Cancer of liver		155	155	C22
	Cancer of larynx		161	161	C32
	Cancer of trachea, bronchus, lung		162	162	C33-4
*Endocrine/*	Nutritional deficiency anaemia		280–1	280–1	D50-3
*nutritional*	Diabetes mellitus (50%)	0–49	250	250	E10-4
	Alcohol and drug related diseases		291, 303-4	291-2, 303-5	F10–6, F18-9
*Cardiovascular*	Ischaemic heart disease (50%)		410–4	410–4	I20-5
	Cerebrovascular disease (50%)		430–8	430–8	I60-9
	Aortic aneurysm		441	441	I71
*Respiratory*	Chronic obstructive pulmonary disease		490–2	490-2, 496	J40-4
*Digestive*	Cirrhosis of liver		571	571	K70, K73-4
*External*	Land transport accidents		E810-27	E810-29, E846-8	V01–4, V06, V09–80, V82–9, V98-9
	Falls		E880-6	E880-6, E888	W00-19
	Fires, burns		E890-9	E890-9	X00-9
	Accidental poisonings		E850-77	E850-69	X40-9
	Drowning		E910	E910	W65-74
	Suicide and self-inflicted injuries		E950-9, E980-9	E950-9, E980-9	X60-84, Y10-34
	Violence		E960-9	E960-9	X85-Y09

Notes.

a The list for preventable causes of death is based on Mühlichen (2019) who relied primarily on Page et al. (2006). We included laryngeal cancer as it is highly associated with tobacco and alcohol consumption, which are important risk factors, particularly in the eastern parts of Germany and Austria (Ahrens et al., 1991; Simonato et al., 1998; Tobias and Jackson, 2001; Phelan et al., 2004). Compared to Mühlichen (2019), we added self-inflicted injuries (events of undetermined intent), as there are substantial differences in coding practices regarding the distinction between suicides and accidents across nations and regions (Danilova et al., 2021).

b In the original ICD-8, HIV/AIDS was not included. However, Switzerland – that still used ICD-8 at the time when the disease spread – added code 137 to their national ICD-8 version.

## Data Availability

The authors do not have permission to share data. The cause-specific mortality data can be requested for a fee at the statistical offices of Austria, Germany, Italy and Switzerland. For Germany, we accessed individual-level cause-of-death statistics at the Research Data Centre of the Federal Statistical Office and the Statistical Offices of the Federal States (DOI: https://doi.org/10.21242/23211.1992.00.00.1.1.0 to https://doi.org/10.21242/23211.2019.00.00.1.1.0).
